# Predicting plaque-gingivitis risk in schoolchildren using an interpretable machine learning model: a cross-sectional study

**DOI:** 10.1186/s12903-025-07245-y

**Published:** 2025-12-15

**Authors:** Linping Wu, Shaochen Su, El-Sayed Salama, Xuexia Ma, Liyuan Chen, Yuanming Wang

**Affiliations:** 1 School of Stomatology, Lanzhou University, 199 Donggang West Road, Lanzhou, 730000 Gansu China; 2 The First Hospital of Lanzhou University, Lanzhou, 730000 Gansu China; 3 School of Public Health, Lanzhou University, Lanzhou, 730000 Gansu China

**Keywords:** Gingivitis, Machine learning, SHAP, Preventive dentistry, Risk stratification, Schoolchildren, Oral health

## Abstract

**Background:**

This study aimed to develop an interpretable machine learning (ML) model for predicting plaque-induced gingivitis risk in schoolchildren using questionnaire data. To enhance the model’s interpretation, SHapley Additive exPlanations (SHAP) method was applied to analyze and explain the risk factors associated with plaque-gingivitis.

**Materials and methods:**

Using multi-stage cluster random sampling, 1755 children aged 6–12 in Lanzhou were enrolled. Participants completed a 22-item questionnaire and underwent clinical dental examinations. The collected data were stratified and randomly divided into a training set (70%) and a testing set (30%), with an independent external validation cohort (*n* = 120) prospectively collected for generalizability assessment. Feature selection was performed using Least Absolute Shrinkage and Selection Operator (LASSO) regression. Six ML algorithms—Light Gradient Boosting Machine (LightGBM), random forest (RF), logistic regression (LR), eXtreme Gradient Boosting (XGBoost), decision tree (DT), and K-nearest neighbors (KNN)—were employed to process the data. The efficacy of each algorithm was evaluated using area under the curve (AUC), sensitivity (recall), specificity, accuracy, precision, F1–score and decision curve analysis. Using the SHAP method, all predictors of gingivitis prevalence in children were ranked by importance.

**Results:**

51.3% (901/1755) of the children were clinically diagnosed with plaque‑induced gingivitis. 11 key predictors were selected using LASSO regression to build the ML models. Among all models, the RF achieved the highest discrimination (training AUC: 0.991; testing AUC: 0.909), followed closely by LightGBM (training AUC: 0.970; testing AUC: 0.904). The RF model was selected as the optimal model and maintained generalizability (external validation AUC: 0.824). SHAP analysis identified key predictors ranked by importance, including brushing frequency, age, regular dental checkups, brushing time, gingival bleeding during brushing, and annual income.

**Conclusion:**

An interpretable RF model accurately stratified gingivitis risk using self-reported factors. This ML-driven strategy may reduce reliance on resource-intensive clinical examinations, supporting scalable pediatric gingivitis prevention in resource-limited settings.

**Supplementary Information:**

The online version contains supplementary material available at 10.1186/s12903-025-07245-y.

## Background

Plaque-induced gingivitis remains the most prevalent periodontal condition affecting children and adolescents globally [1]. Prevalence shows significant regional variation, ranging from 23% to 77% among Latin American adolescents to 38.6% in Chinese children aged 6 to12 [2, 3]. Many patients overlook the condition due to absent pain and rare spontaneous bleeding [4, 5]. Critically, untreated gingivitis may advance to periodontitis along a pathological continuum characterized by connective tissue destruction and osseous resorption [1, 4], a progression driven by a dysbiotic shift in the subgingival microbiome and an exaggerated host inflammatory response [6]. Effective prevention of plaque-induced gingivitis is a critical strategy for mitigating periodontitis risk [4, 7]. Potential risk factors encompass overweight status, type of diet, oral hygiene practices, and socioeconomic status [8–10]. Additionally, circumpubertal elevations in estrogen and progesterone enhance gingival susceptibility to local irritants, thereby amplifying inflammatory responses and increasing gingivitis risk [3, 7, 11, 12]. The 6–12-year, period of mixed dentition, immediately precedes circumpuberty, when hormonal changes and shifting behaviors can amplify plaque accumulation and gingival inflammatory responses [2, 3]. Consequently, identifying high-risk children prior to adolescence provides a tractable window for durable behavior change and prevention.

Conventional periodontal assessments demand substantial time and resources, rendering population-level screening impractical with standard oral examination methods [13]. This necessitates scalable screening tools, particularly for pediatric populations where early intervention is crucial. Machine learning (ML) algorithms offer a resource-efficient alternative, leveraging pattern recognition in complex datasets to overcome scalability barriers [14–16]. Self-reported questionnaires combined with ML have demonstrated potential in assessing dental risks [14], yet no models exist for assessing gingivitis risk specifically in 6–12-year-olds using brief, self-administered questionnaires. Furthermore, challenges remain in this field. First, given the varying effectiveness of different ML algorithms, evaluating and comparing their performance is essential [17, 18]; second the inherent “black-box” nature of many ML models impedes clinical trust and adoption by raising concerns about interpretability [17, 19]. The SHapley Additive exPlanations (SHAP) method offers a promising solution to enhance transparency and clinical interpretability by integrating game-theoretic credit allocation with localized interpretability, generating clinically actionable visualizations that quantify variable-specific risk contributions [19, 20].

Therefore, this study aimed to develop and validate an interpretable ML model specifically for children aged 6–12 years using self-reported variables to assess gingivitis risk, with SHAP methodology employed to enhance clinical interpretability and inform targeted screening strategies for high-risk individuals.

## Methods

### Study population

The research dataset finally included 1755 children aged 6 to 12 years, collected from 3 June 2024 to 28 November 2024 in Lanzhou, China. An independent validation cohort (*n* = 120) was subsequently recruited from a later period (temporal validation) from 3 March 2025 to 28 March 2025. Following the completion of data collection and organization, the final analytical dataset was locked on 20 April 2025, after which all ML modeling and statistical analyses were performed. The cross-sectional study employed a stratified multi-stage cluster random sampling across districts, schools, and classes to ensure population representativeness. Ethical approval (No. 2024/072) was granted by the Lanzhou University School of Stomatology, with written informed consent obtained from their family after being informed about the study details. All procedures adhered to the Declaration of Helsinki.

**Fig. 1:**
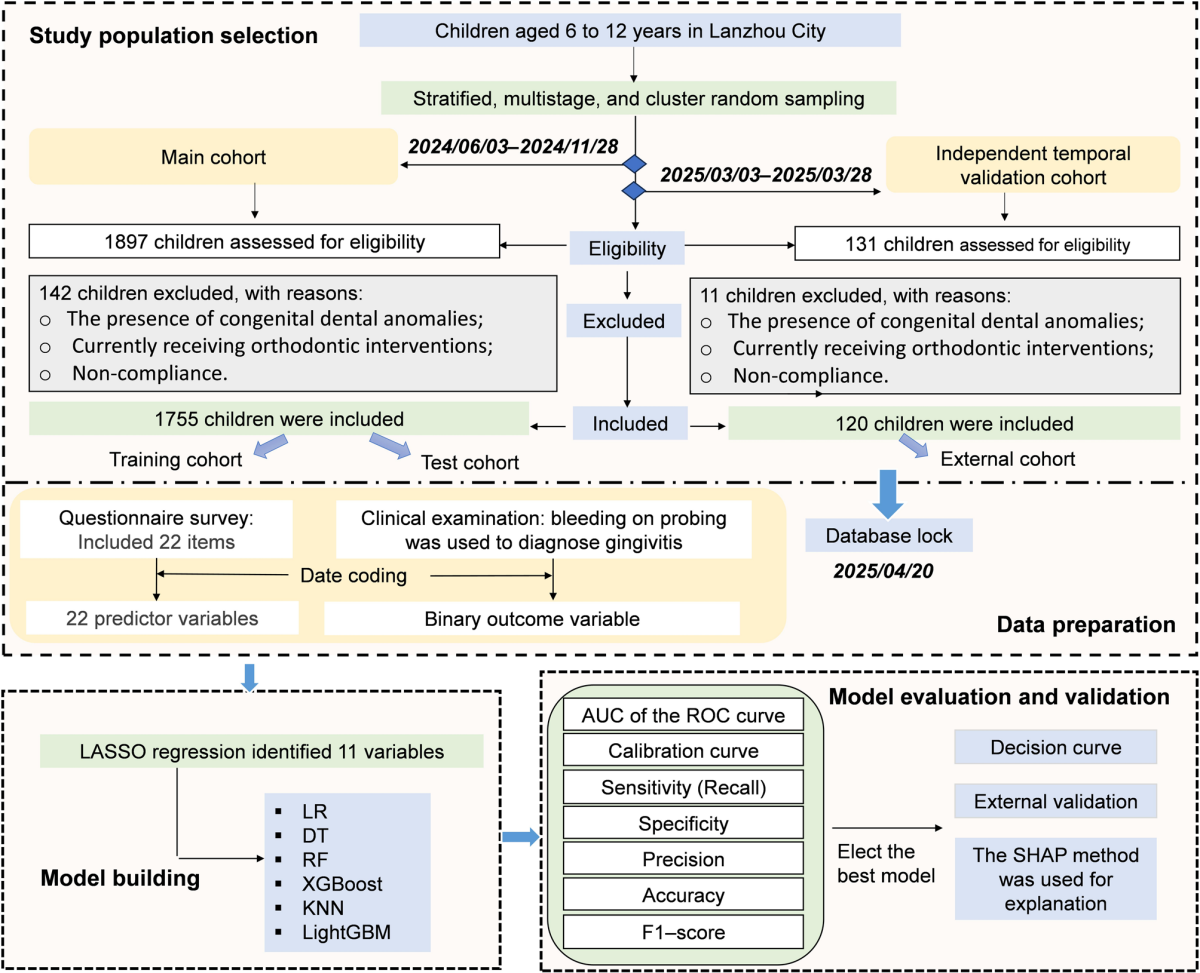
Study flow chart

Children were eligible for the study if they met all four criteria: (1) aged 6 to12; (2) residing in Lanzhou City for more than 6 months; (3) full eruption of all permanent first molars; and (4) being capable of cooperating and completing the clinical examination. Exclusion criteria comprised: (1) the presence of congenital dental anomalies; (2) currently receiving orthodontic interventions; (3) incomplete eruption of any first molar; (4) non-compliance with the examination despite psychological and behavioral guidance. The flowchart of this study is shown in Fig. 1.

### Data collection and preprocessing

A 22-item questionnaire was administered to children and parents to assess gingivitis-related factors. It was developed based on the epidemiologic assessment of periodontal diseases developed by the World Health Organization (WHO) [21], with modifications informed by other relevant studies [3, 22] to ensure contextual appropriateness. Prior to the main study, the questionnaire’s face and content validity were established. It was reviewed by a panel of three pediatric dentists and one epidemiologist, yielding a scale-level Content Validity Index ranging from 0.83 to 0.94. Furthermore, a pilot test involving nine schoolchildren and their parents confirmed the questionnaire’s clarity and feasibility. All individuals involved in these preliminary phases were excluded from the final study cohort. It contained questions including: (1) demographic background (participant age, biological sex, residence area, single child status, and caregiver educational attainment); (2) oral hygiene awareness (gingival bleeding during brushing is normal, brushing prevents bleeding gums); (3) oral behavioral patterns (sugar intake frequency, daily brushing occurrences, brushing time, bleeding from brushing, floss daily, fluoride toothpaste use, rinsing after meals, toothbrush change frequency, parents would supervise tooth brushing every day and regular dental examinations), and (4) undesirable oral habits (unilateral chewing and mouth breathing). The full questionnaire used in this study is available in Supplementary Material 1.

Periodontal assessments employed standardized probing techniques where the UNC-15 probe tip was delicately inserted into the gingival sulcus to explore its complete depth. Using a controlled pressure (≤ 25 g), the instrument was carefully moved in vertical motions along the root surface’s anatomical contours to check for bleeding [4, 9]. Each tooth (excluding second/third molars) was assessed at six sites (mesiobuccal, buccal, distobuccal, disto-oral, oral and mesio-oral). BOP was recorded dichotomously (present/absent) within 10 s per site. The percentage of bleeding sites (BOP%) was calculated per individual. According to the new criteria published by European Federation of Periodontology/American Academy of Periodontology (EFP/AAP) in 2018, gingivitis is defined as 10% or more of sites with positive bleeding on probing score (BOP% ≥ 10%) with probing depths ≤ 3 mm [9]. Three examiners were trained and calibrated against a gold-standard periodontist using nine children not included in the final study, until an inter-examiner kappa score of >0.85 was consistently achieved.

All the information was extracted from the questionnaires and the oral health examination. Categorical variables were encoded: ordinal features (e.g., brushing frequency) assigned integer levels; nominal variables (e.g., residence) one-hot encoded.

### Feature selection

This study employed a stratified random split to divide the data into a training set (70%) and an independent test set (30%), maintaining the distribution of the target variable (gingivitis prevalence) to be consistent with the entire data set. To address potential multicollinearity and identify the most relevant predictors from the initial set of variables, Least Absolute Shrinkage and Selection Operator (LASSO) regression was used in training set with the *‘glmnet’* package in R. Features selected by LASSO regression were retained for subsequent model development.

### Least Absolute Shrinkage and Selection Operator

LASSO regression is a regularization technique that applies an L1 penalty to the regression coefficients, compressing coefficients of less important features towards zero, effectively filtering out non-informative variables and retaining those with substantial contributions to the prediction model [23]. This promotes model sparsity and interpretability while mitigating overfitting. The optimal regularization strength, controlled by the lambda (λ) parameter, was usually determined using 10-fold cross-validation [24]. The *‘one-standard-error’* (1-SE) rule was applied to select the most parsimonious model (lambda.1se) within one standard error of the lambda that gave the minimum mean cross-validated error [23–25]. This approach balances model complexity with predictive performance. In practice, this rule favors a simpler model by excluding features with less stable contributions, thereby reducing the risk of overfitting and improving generalizability, while maintaining comparable predictive accuracy [23, 24].

###  Model development and evaluation

In this study, six common ML methodologies were adapted to develop the screening model: logistic regression (LR), decision trees (DT), K-nearest neighbors (KNN), Light gradient boosting machine (LightGBM), random forests (RF), and eXtreme gradient boosting (XGBoost) algorithms. LR was chosen because of its proven effectiveness in dichotomous outcome forecasting and high interpretability [26]. The application of multiple machine learning methods leverages their distinct strengths: DT provides clarity in reasoning and ease of mapping the decision-making process through hierarchical structures; RF demonstrates effectiveness in managing high-dimensional datasets; KNN offers a simple implementation and is effective at identifying localized patterns through proximity-based analysis; LightGBM is particularly suitable for large-scale datasets and provides an intuitive method for assessing feature importance; XGBoost introduces regularization to control model complexity, effectively preventing overfitting [18, 24, 27]. The ML models were implemented using the following R packages: ‘*randomForest*’ for RF, ‘*lightgbm*’ for LightGBM, ‘*glm*’ for LR, ‘*xgboost*’ for XGBoost, ‘*rpart*’ for DT, and ‘*kknn*’ for KNN.

Hyperparameter tuning employed the grid search method (R ‘*caret*’ package) with 5-fold cross-validation to select configurations to maximize the mean area under the curve (AUC) of receiver operating characteristic (ROC). The hyperparameter values for each algorithm are presented in Supplementary Material 2.

**Fig. 2:**
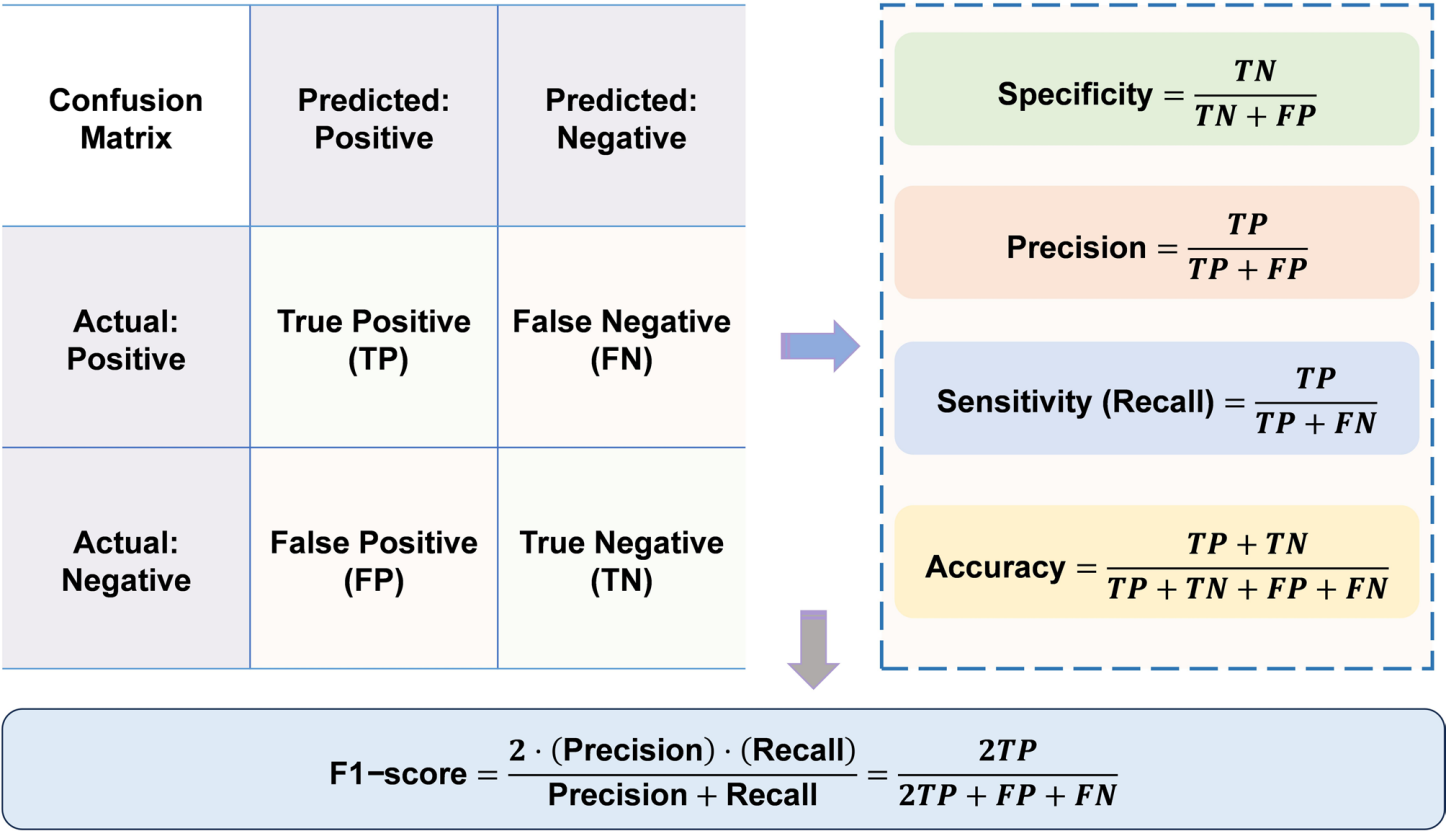
Confusion matrix plot and evaluation index for the models

Model performance was comprehensively evaluated using multiple metrics: (1) AUC-ROC to assess discriminative ability in distinguishing high-risk from low-risk individuals [25]; (2) calibration curves to evaluate the agreement between predicted probabilities and observed outcomes, ensuring risks [25, 27]; (3) key classification metrics derived from the confusion matrix (Fig. 2 shows the equations for evaluation metrics used), including sensitivity (recall), specificity, precision, accuracy and F1-score. The statistical significance of the difference in AUC between the top-performing models was assessed using DeLong’s test on the test set. The optimal model’s decision curve analysis (DCA) was plotted to quantify the model’s clinical utility across varying risk thresholds [28]. Furthermore, external validation was conducted to verify its generalizability across diverse populations [25, 29].

### Model interpretation

To interpret the predictions of the optimal model identified and gain insights into the key drivers of gingivitis prediction, SHAP analysis was performed.

### SHapley Additive exPlanations

SHAP method is a unified approach based on cooperative game theory and provides a unified framework for explaining the output of ML model [30, 31]. They quantify the contribution of each feature to the prediction for an individual instance relative to the average prediction (baseline), offering both local (per-instance) and consistent interpretations of model behavior [19, 32].

The R package *‘shapviz’* and *‘kernelshap’* were utilized for SHAP computation and visualization. Global feature importance was assessed by calculating the mean absolute SHAP value (|SHAP|) for each feature across the entire test dataset. The direction (positive or negative) and magnitude of each feature’s influence on the predicted probability of gingivitis were visualized using SHAP summary plots (bee swarm plots). Local interpretability was demonstrated by analyzing SHAP force plots for two representative individual cases, illustrating how specific feature values contributed to the model’s prediction for those particular children. The workflow was executed using the R software (version: 4.2.0).

## Results

### Baseline characteristics

The final cohort comprised 1,755 children aged 6–12, with gingivitis prevalence at 51.3% (901 cases). The baseline characteristics **(**Table 1) identified significant differences between gingivitis and non-gingivitis groups in age, only-child status, body mass index (BMI), parental education levels, and annual family income (*p* < 0.05). No significant difference was found based on urban-rural residence (*p* > 0.05). Significant differences were also observed for risk factors, including the daily frequency of tooth brushing, brushing time (duration), bleeding from brushing, floss daily, fluoride toothpaste use, rinsing after meals, regular annual dental checkups, frequency of sugar consumption, unilateral chewing, and mouth breathing (*p* < 0.05).

**Table 1:** Comparison of baseline characteristics in the gingivitis and normal groups in children aged 6–12

Variable	Code	*N* (1755)	Normal (*n* = 854) *n* (%)	Gingivitis (*n* = 901) n (%)	*p* - value
Socio-demographic characteristics					
Age (year)					< 0.001
6	6	228 (13.0%)	162 (19.0%)	66 (7.3%)	
7	7	249 (14.2%)	168 (19.7%)	81 (9.0%)	
8	8	252 (14.4%)	131 (15.3%)	121 (13.4%)	
9	9	259 (14.8%)	81 (9.5%)	178 (19.8%)	
10	10	258 (14.7%)	87 (10.2%)	171 (19.0%)	
11	11	257 (14.6%)	92 (10.8%)	165 (18.3%)	
12	12	252 (14.4%)	133 (15.6%)	119 (13.2%)	
Sex					0.933
Male	0	868 (49.5%)	421 (49.3%)	447 (49.6%)	
Female	1	887 (50.5%)	433 (50.7%)	454 (50.4%)	
Area					0.910
Urban	0	1033 (58.9%)	501 (58.7%)	532 (59.0%)	
Rural	1	722 (41.1%)	353 (41.3%)	369 (41.0%)	
BMI					0.006
Underweight	0	93 (5.3%)	39 (4.6%)	54 (6.0%)	
Normal	1	1299 (74.0%)	663 (77.6%)	636 (70.6%)	
Obesity	2	298 (17.0%)	121 (14.2%)	177 (19.6%)	
Overweight	3	65 (3.7%)	31 (3.6%)	34 (3.8%)	
If only child					< 0.001
Yes	0	501 (28.5%)	163 (19.1%)	338 (37.5%)	
No	1	1254 (71.5%)	691 (80.9%)	563 (62.5%)	
Father’s education level					< 0.001
≤ 9 years	0	427 (24.3%)	82 (9.6%)	345 (38.3%)	
10–12 years	1	317 (18.1%)	138 (16.2%)	179 (19.9%)	
≥ 13 years	2	1011 (57.6%)	634 (74.2%)	377 (41.8%)	
Mother’s education level					< 0.001
≤ 9 years	0	385 (21.9%)	73 (8.5%)	312 (34.6%)	
10–12 years	1	330 (18.8%)	42 (16.6%)	188 (20.9%)	
≥ 13 years	2	1040 (59.3%)	639 (74.8%)	401 (44.5%)	
Annual family income (yuan)					< 0.001
≤ 50,000	0	148 (8.4%)	47 (5.5%)	101 (11.2%)	
50,000–100,000	1	808 (46.0%)	323 (37.8%)	485 (53.8%)	
100,000–200,000	2	509 (29.0%)	272 (31.9%)	237 (26.3%)	
200,000–400,000	3	203 (11.6%)	145 (17.0%)	58 (6.4%)	
≥ 400,000	4	87 (5.0%)	67 (7.8%)	20 (2.2%)	
Oral health behaviors					
Brushing frequency					< 0.001
Twice a day or more	0	791 (45.0%)	574 (67.2%)	217 (24.1%)	
Once a day	1	747 (42.6%)	277 (32.4%)	470 (52.2%)	
Not everyday	2	217 (12.4%)	3 (0.4%)	214 (23.8%)	
Brushing time					< 0.001
≥ 3 min	0	260 (14.8%)	221 (25.9%)	39 (4.3%)	
1–3 min	1	1148 (65.4%)	563 (65.9%)	585 (64.9%)	
≤ 1 min	2	347 (19.8%)	70 (8.2%)	277 (30.7%)	
Floss daily					< 0.001
Yes	0	117 (6.7%)	93 (10.9%)	24 (2.7%)	
No	1	1638 (93.3%)	761 (89.1%)	877 (97.3%)	
Fluoride toothpaste use					0.439
Yes	0	648 (36.9%)	307 (35.9%)	341 (37.8%)	
No	1	1107 (63.1%)	547 (64.1%)	560 (62.2%)	
Rinsing after meals					< 0.001
Yes	0	261 (14.9%)	174 (20.4%)	87 (9.7%)	
No	1	1494 (85.1%)	680 (79.6%)	814 (90.3%)	
Toothbrush change frequency					0.057
≤ 3 months	0	957 (54.5%)	486 (56.9%)	471 (52.3%)	
>3 months	1	798 (45.5%)	368 (43.1%)	430 (47.7%)	
Bleeding from brushing					< 0.001
Never	0	811 (46.2%)	490 (57.4%)	321 (35.6%)	
Sometimes	1	782 (44.6%)	335 (39.2%)	447 (49.6%)	
Frequently	2	162 (9.2%)	29 (3.4%)	133 (14.8%)	
Your parents will supervise your tooth brushing every day					0.956
Yes	0	487 (27.7%)	238 (27.9%)	249 (27.6%)	
No	1	1268 (72.3%)	616 (72.1%)	652 (72.4%)	
Regular annual dental checkups					< 0.001
Yes	0	543 (30.9%)	399 (46.7%)	144 (16.0%)	
No	1	1212 (69.1%)	455 (53.3%)	757 (84.0%)	
Consumption of sugars and sweet foods					< 0.001
Once a day or more	0	333 (19.0%)	170 (19.9%)	163 (18.1%)	
Two to six times a week	1	940 (53.6%)	519 (60.8%)	421 (46.7%)	
Once a week or less	2	482 (27.5%)	165 (19.3%)	317 (35.2%)	
Oral health knowledge					
Bleeding from brushing is normal					0.001
Yes	0	760 (43.3%)	262 (30.7%)	498 (55.3%)	
No	1	462 (26.3%)	386 (45.2%)	76 (8.4%)	
Unclear	2	533 (30.4%)	206 (24.1%)	327 (36.3%)	
Brushing prevents bleeding gums					0.001
Yes	0	369 (21.0%)	251 (29.4%)	118 (13.1%)	
No	1	698 (39.8%)	223 (26.1%)	475 (52.7%)	
Unclear	2	688 (39.2%)	380 (44.5%)	308 (34.2%)	
Oral bad habits					
Unilateral chewing					0.896
Yes	0	301 (17.2%)	148 (17.3%)	153 (17.0%)	
No	1	1454 (82.8%)	706 (82.7%)	748 (83.0%)	
Mouth breathing					0.001
Yes	0	300 (17.1%)	81 (9.5%)	219 (24.3%)	
No	1	1455 (82.9%)	773 (90.5%)	682 (75.7%)	

### Feature selection

**Fig. 3:**
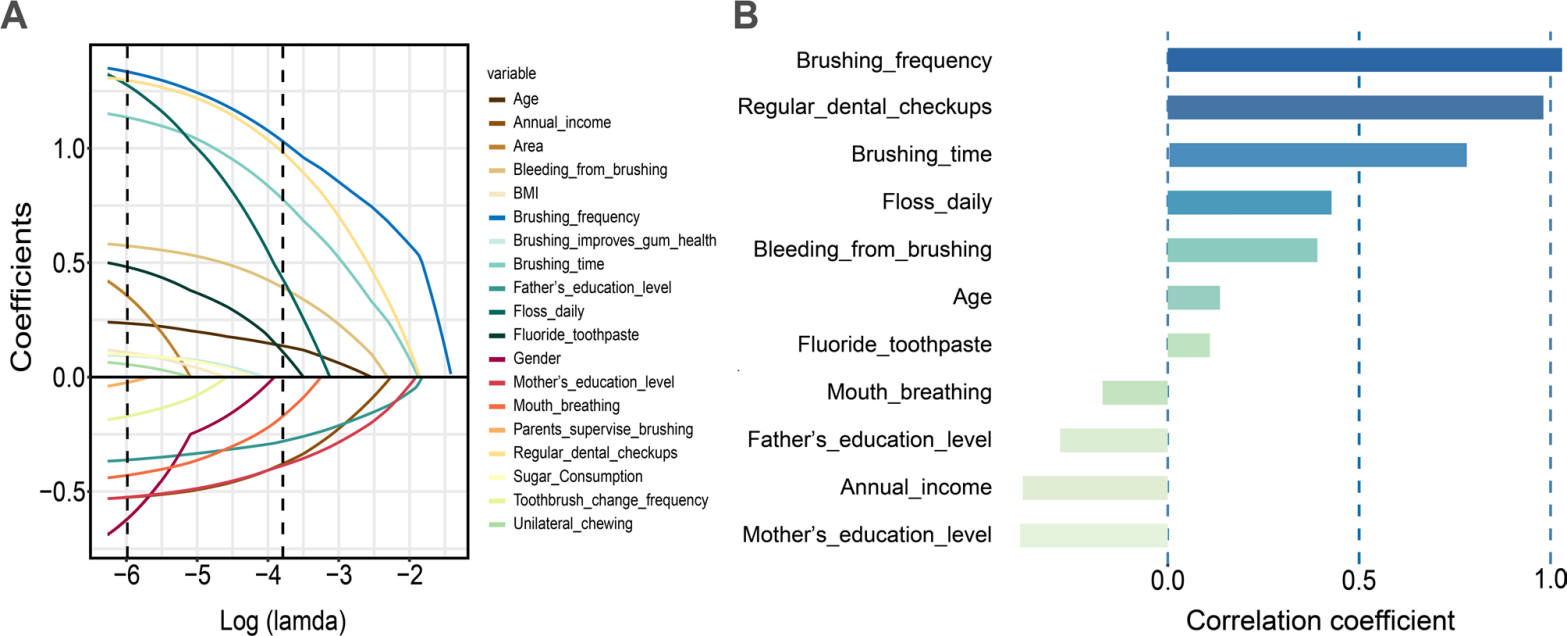
Feature selection via LASSO regression. **A**. LASSO coefficient profiles of the 22 features. **B**. The coefficient values of the variables retained in LASSO regression

The data cohort was randomly partitioned into a training (gingivitis group, 630/1228, 51%) and a test (gingivitis group, 271/527, 51%) subsets in a 7:3 ratio. Feature identification for the training cohort was conducted by applying LASSO regression and a coefficient profile plot was produced against the log(lambda) sequence (Fig. 3A). Employing 10-fold cross-validation with the 1-SE criterion (Supplementary Material 3), the optimal regularization strength was set at λ = 0.02261307. This process identified 11 variables significantly associated with gingivitis risk for inclusion in the predictive models: age, father’s education level, mother’s education level, annual income, brushing frequency, brushing time, floss daily, fluoride toothpaste, bleeding from brushing, regular dental checkups, mouth breathing **(**Fig. 3B).

### Model building and evaluation

**Fig. 4:**
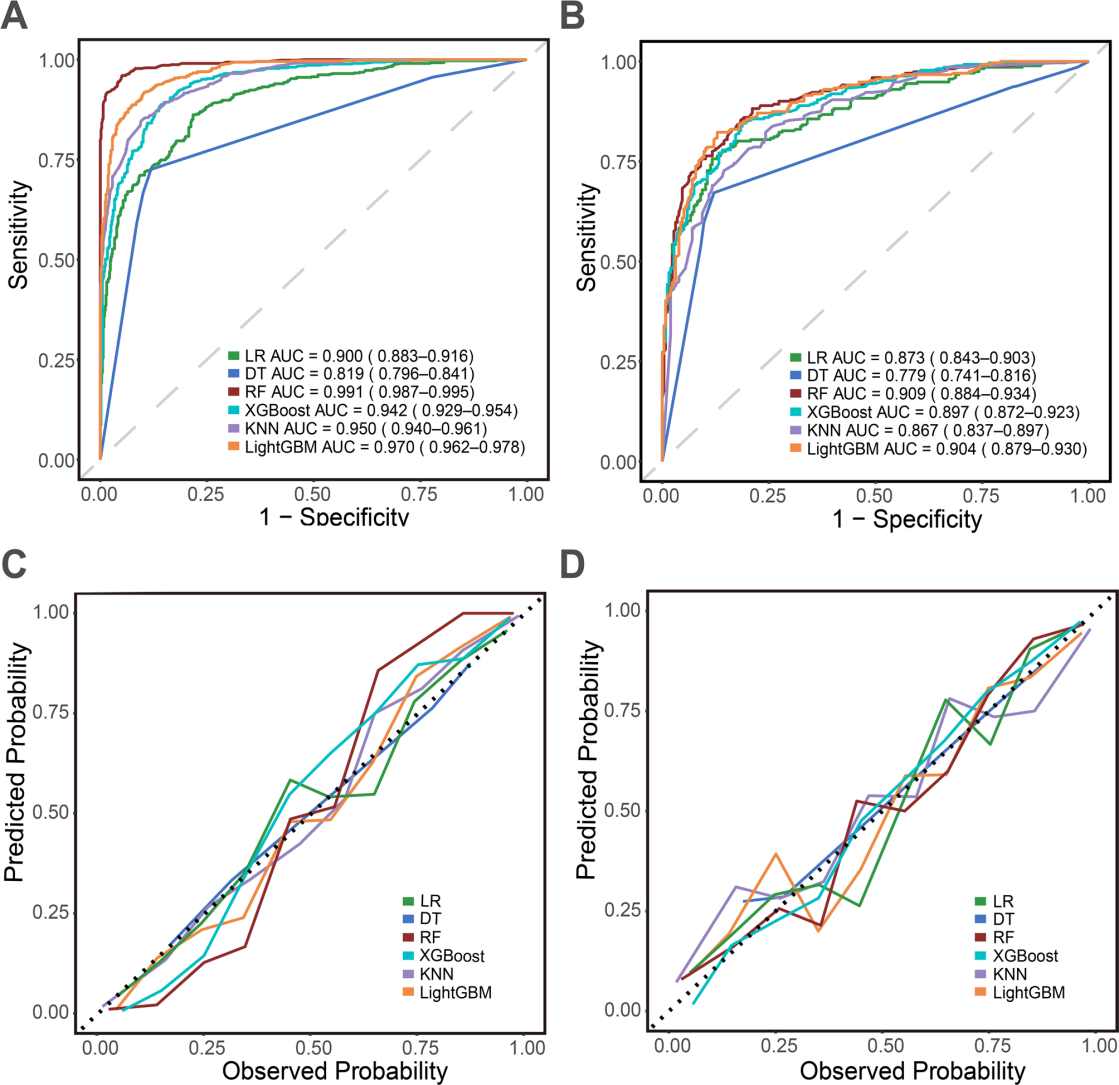
ML models comprehensive analysis. **A**. ROC curves for the training set. **B**. ROC curves for the testing set. **C**. Calibration curves for the training set. **D**. Calibration curves for the testing set

All six models demonstrated robust discriminatory capacity, except for the DT classifier which showed lower performance (training: 0.819, 95% CI: 0.796–0.841; testing: 0.779, 95% CI: 0.741–0.816) (Fig. 4A and B; Table 2). This lower performance is likely due to its nature as a single-tree model, which is more susceptible to overfitting and its higher variance compared to ensemble methods [18, 24, 27]. RF yielded the highest AUC (training: 0.991, 95% CI: 0.987–0.995; testing: 0.909, 95% CI: 0.884–0.934) among all models, followed closely by LightGBM (training: 0.970, 95% CI: 0.962–0.978; testing: 0.904, 95% CI: 0.879–0.930). A DeLong’s test performed on the test set confirmed that the difference in AUC between the top two models (RF and LightGBM) was not statistically significant (*p* = 0.78).

The calibration curves of all models displayed favorable agreement between predicted probabilities and observed outcomes in both the training and testing sets (Fig. 4C and D).

Table 2 summarizes evaluation metrics. In training cohort, the RF model the achieved highest sensitivity (0.943), specificity (0.958), precision (0.950) and accuracy (0.960), F1–score (0.951). Both RF and LightGBM demonstrated excellent performance across key metrics in the testing cohort.

**Table 2:** Performances of the ML models for screening gingivitis in children aged 6–12

Model	Data sets	AUC (95% CI)	Sensitivity	Specificity	Accuracy	Precision	F1–score
LR	Training set	0.900 (0.883–0.916)	0.789	0.826	0.807	0.827	0.808
	Testing set	0.873 (0.843–0.903)	0.775	0.832	0.803	0.830	0.802
LightGBM	Training set	0.970 (0.962–0.978)	0.902	0.903	0.902	0.907	0.904
	Testing set	0.904 (0.879–0.930)	0.823	0.856	0.839	0.858	0.840
DT	Training set	0.819 (0.796–0.841)	0.725	0.881	0.801	0.866	0.789
	Testing set	0.779 (0.741–0.816)	0.672	0.879	0.772	0.855	0.752
RF	Training set	0.991 (0.987–0.995)	0.943	0.958	0.950	0.960	0.951
	Testing set	0.909 (0.884–0.934)	0.804	0.848	0.825	0.848	0.826
XGBoost	Training set	0.942 (0.929–0.954)	0.849	0.878	0.863	0.880	0.864
	Testing set	0.897 (0.872–0.923)	0.786	0.840	0.812	0.839	0.811
KNN	Training set	0.950 (0.940–0.961)	0.860	0.878	0.869	0.868	0.846
	Testing set	0.867 (0.837–0.897)	0.809	0.757	0.782	0.686	0.719

**Fig. 5:**
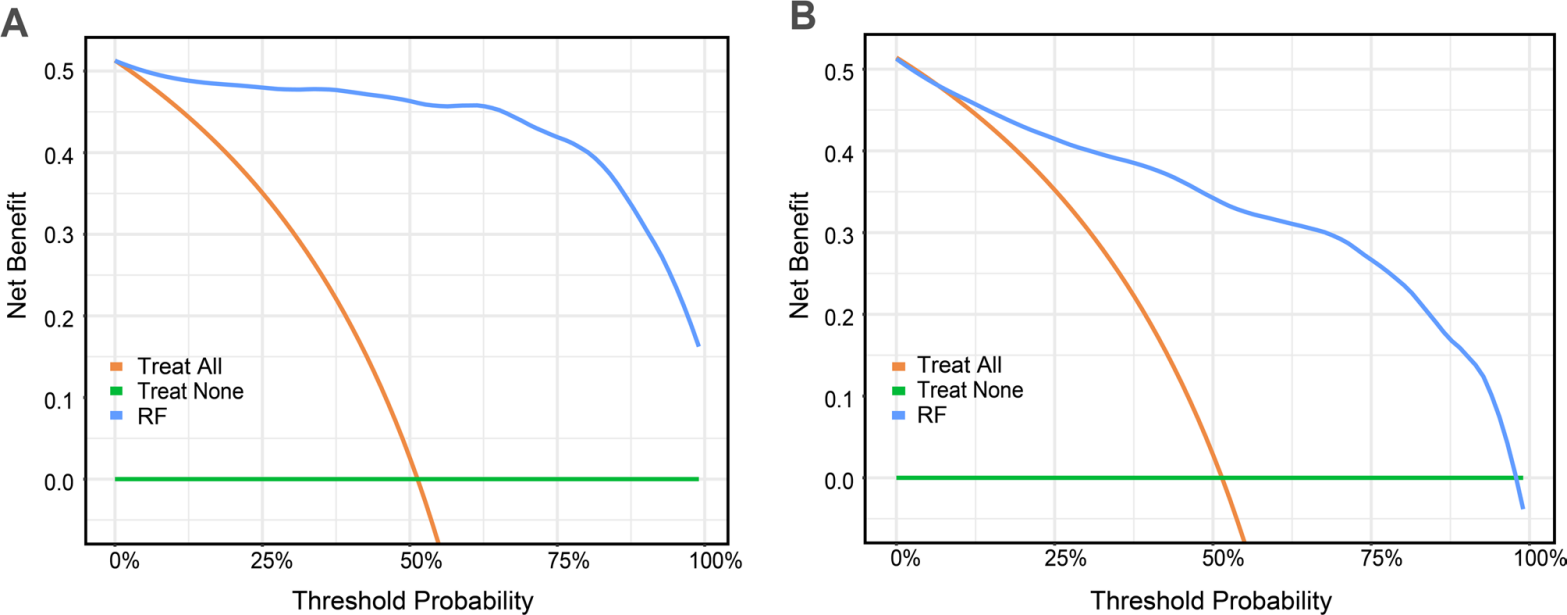
DCA curves of the RF model. **A** DCA curve for the training set. **B **DCA curve for the testing set

The RF model was selected as the final optimal model. This decision was grounded not only in its top test AUC but also in its superior computational efficiency and the inherent robustness of its ensemble structure for generating stable, interpretable outputs, both of which are vital for the intended public health application [33, 34]. The results of the DCAs (Fig. 5) revealed that the mean net benefits of the RF model for predicting gingivitis were superior to the default approaches (treating all patients or none) throughout a broad spectrum of threshold probabilities. This demonstrates significant clinical value for identifying high-risk pediatric gingivitis cases in children aged 6 to 12 using our RF model.

**Fig. 6:**
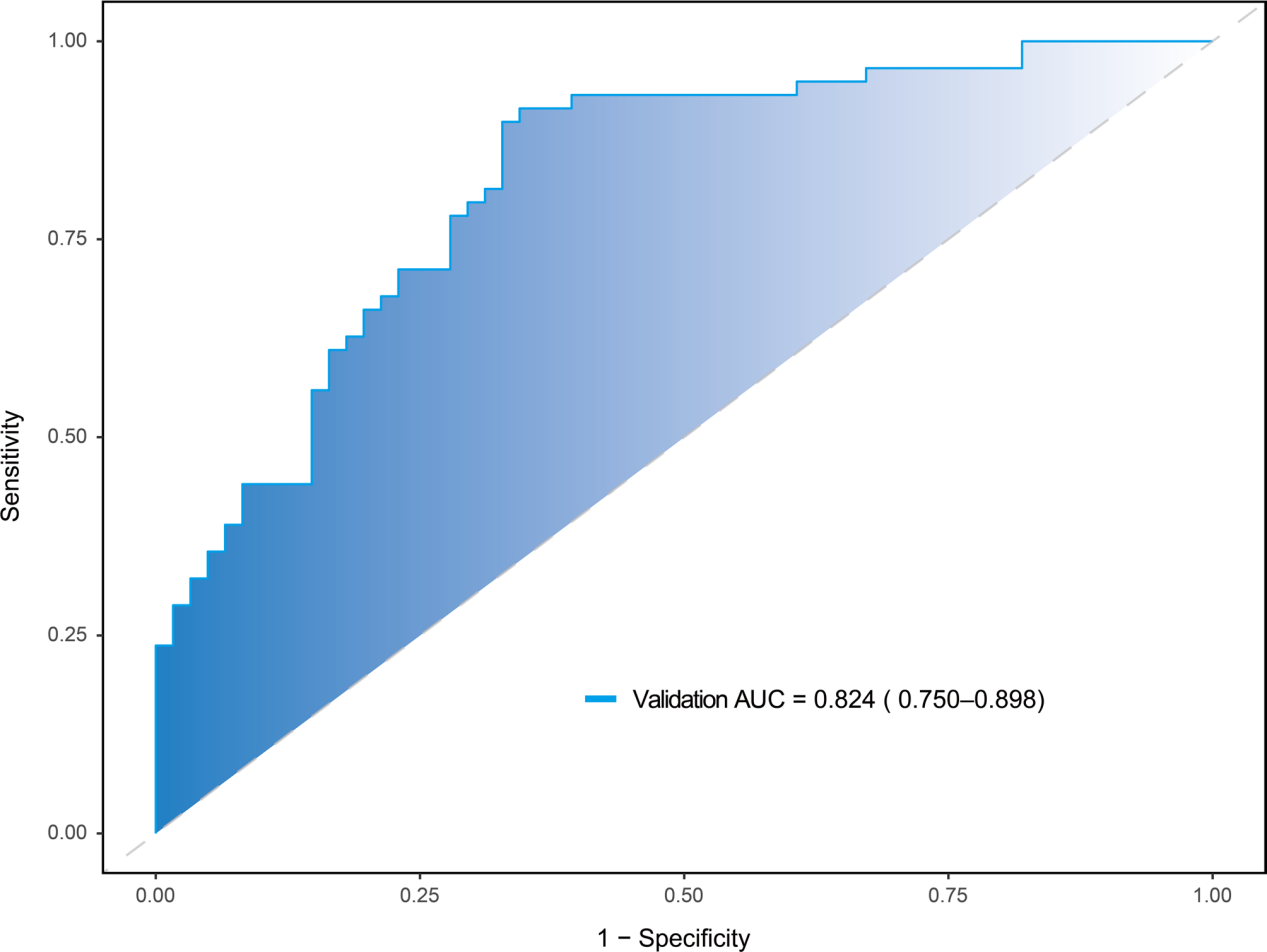
The ROC curve of the RF model in the validation set

The temporal validity of the optimal RF model was assessed on an independent cohort (Baseline characteristics in (Supplementary Material 4). This validation demonstrated robust performance, achieving an AUC of 0.824 (95% CI: 0.750–0.898), as illustrated in Fig. 6.

### Interpretability analysis

**Fig. 7:**
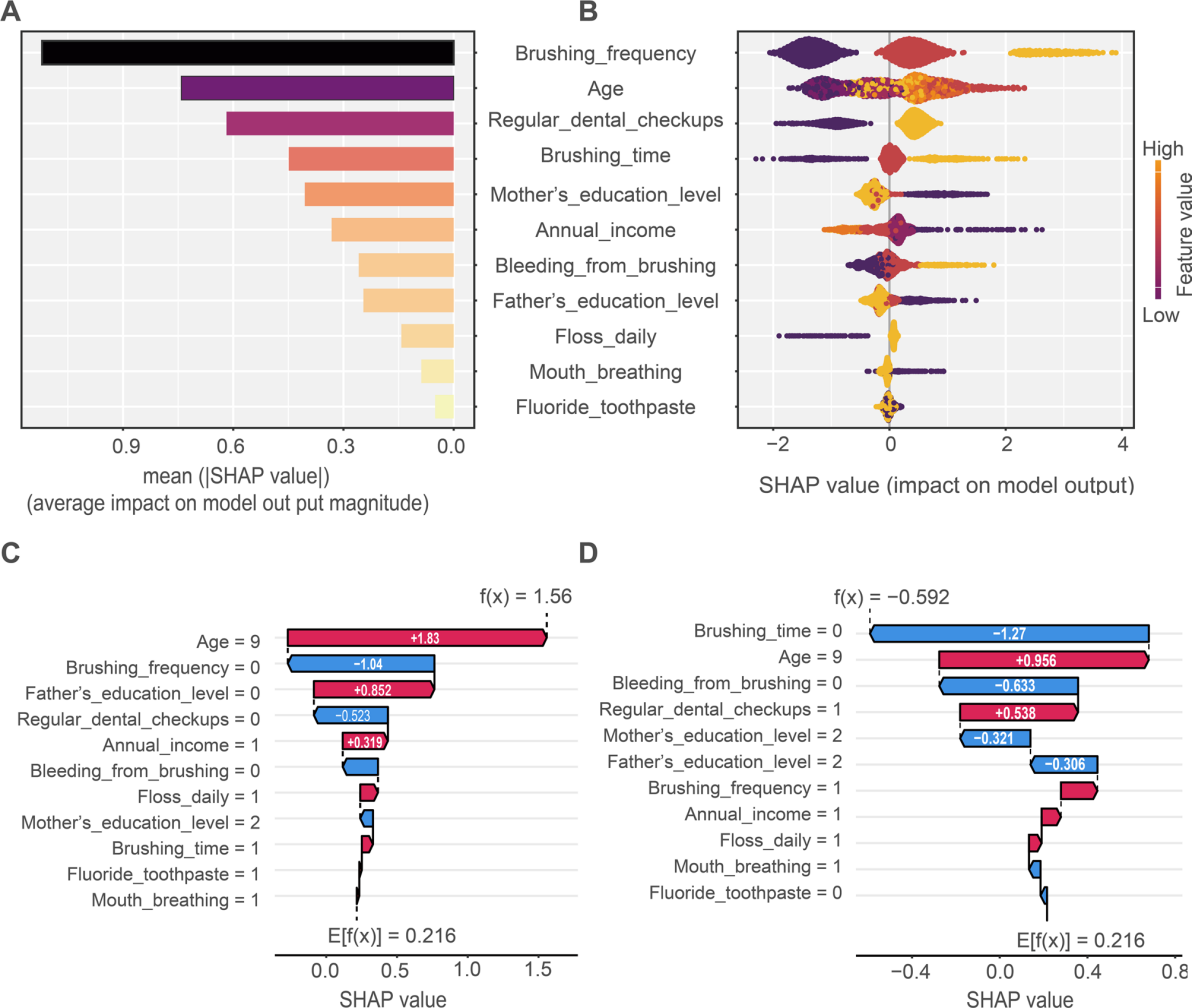
Visually interpret the RF model using SHAP. **A**. Predictor importance ranking; **B**. SHAP summary plot. **C** SHAP force plot for one selected child diagnosed with gingivitis. **D** SHAP force plot for one selected child diagnosed with non-gingivitis

The SHAP analysis quantified variable importance and directional effects for the RF model. The variable importance chart (Fig. 7A) ranked brushing frequency as the greatest predictor. Subsequent influential variables comprised age, frequency of dental checkups, brushing time, mother’s educational level, annual income, and father’s education level. Furthermore, SHAP values were employed to quantify the directional effects of features on the predicted risk of gingivitis **(**Fig. 7B). Each point represents an individual patient. The color indicates the feature value, with red corresponding to higher values and blue to lower values. The position on the x-axis shows the impact on the prediction (positive SHAP value increases risk, negative decreases risk). Key findings include: lower brushing frequency, older age, shorter brushing time, and frequent bleeding during brushing were consistently associated with an increased predicted risk of gingivitis. In contrast, regular dental checkups, higher annual family income, elevated parental education levels, and the daily use of dental floss were associated with a decreased predicted risk.

Figure 7 presents SHAP force plots for two representative individual cases: (C) a child diagnosed with gingivitis (true positive) and (D) a child without gingivitis (true negative). These plots illustrate how each feature’s value shifts the model’s prediction away from the baseline value (the average model output for the entire dataset). Features shown in red increase the predicted risk of gingivitis, while those in blue decrease it. For the high-risk child in Fig. 7C, targeted advice could be directed toward improving oral hygiene behaviors, such as increasing brushing time and incorporating daily flossing. In contrast, for the child shown in Fig. 7D, the model indicates that existing behaviors are already protective, suggesting that reinforcing current routines may be sufficient.

## Discussion

In this study, we developed an interpretable ML model based on self-reported questionnaires that accurately stratified the risk of gingivitis in schoolchildren. The RF model demonstrated the best performance (training AUC: 0.991; testing AUC: 0.909). While the notably high training AUC could suggest a risk of overfitting, the model’s robust performance on the test set and external validation cohort (AUC: 0.824) confirms its good generalizability. This advantage can be attributed to its ensemble learning framework with bagging and feature randomization, which effectively mitigate overfitting while enhancing generalization capability [35, 36]. This feature constitutes a critical advantage for community-level screening tools operating in highly variable data environments, while simultaneously aligning with our core design principle: reducing dependence on specialized clinical assessments. While dental plaque and calculus are established gingivitis risk factors, their inclusion would require professional examination (via indices like the Plaque Index), contradicting the model’s objective of resource-efficient scalability [3, 4, 7, 11]. By deliberately excluding these clinically intensive variables and instead leveraging clinically validated proxy indicators (e.g., self-reported brushing frequency and gingival bleeding), we maintain scientific rigor while ensuring the tool’s feasibility for population-level deployment.

Through the application of SHAP analysis, several key predictors of gingivitis in children aged 6 to 12 years were identified. Notably, the frequency and duration of toothbrushing emerged as critical variables, corroborating global evidence that suboptimal oral hygiene directly exacerbates plaque accumulation, consequently increasing the risk of gingivitis [3, 7, 9, 37]. Age also emerged as a key indicator, as younger children exhibited relatively better periodontal status compared to their older counterparts [12, 38]. Professional dental visits boost the likelihood of early identification, prevention, and management of oral diseases [5, 11]. We found that regular dental examinations served as a significant screening variable, further emphasizing the critical role of routine dental care in mitigating the incidence of oral diseases. Current findings indicated notable correlations between parental education levels and household income with the risk of gingivitis. These socioeconomic factors likely influence the availability of oral health services and the degree of oral health consciousness, thereby highlighting the imperative for targeted interventions within underserved communities [5, 7]. Gingival bleeding from brushing was identified as a key predictor, which may not only reflect inadequate oral hygiene awareness but also represent a direct manifestation of existing gingival inflammation, serving as a proximate indicator of the disease state [12, 39–41]. The self-assessment of gingival bleeding could serve as an effective strategy for monitoring periodontal health and raising awareness of periodontal disease. Flossing is an essential component of oral hygiene, and research indicates that its effectiveness is significantly enhanced when practiced in conjunction with tooth brushing, thereby optimizing the overall oral environment [42–44]. Furthermore, mouth breathing was found to have a significant association with gingivitis, presumably due to its contribution to a dry oral environment that exacerbates inflammatory responses [45]. The incorporation of fluoride toothpaste usage in the final RF model is noteworthy. Although direct evidence of fluoride’s inhibitory effect on gingival inflammation is limited, some studies indicate that fluoride may reduce gingival inflammation indirectly by inhibiting pathogenic bacteria in the dental plaque biofilm [46–48]. While this secondary preventive effect is relatively modest (ranked 11th in feature importance), it aligns with current oral hygiene recommendations and demonstrates the model’s ability to identify subtle but clinically significant associations.

By quantifying individualized risk contributions, SHAP-based explanations can guide personalized oral hygiene interventions for high-risk children. For instance, a child with low brushing frequency and bleeding gums (high-risk features) could be prioritized for targeted oral hygiene education, while those with regular dental checkups (protective factor) may require less intensive screening. And this provides a feasible framework for implementing a practical two-stage screening strategy within school-based programs. First, parents assist children in completing a school-distributed electronic questionnaire, with the trained model automatically stratifying children into risk categories in real-time. Second, only those identified as “high risk” would undergo a confirmatory clinical examination by a visiting dental professional. This targeted approach prioritizes the allocation of limited clinical resources to those most in need, potentially enabling large-scale screening with minimal specialist time.

Various investigations have used ML methodologies to evaluate periodontal health. For instance, a study utilized LR to predict gingival bleeding among children (12-year-old) in Sichuan Province, China, which achieved an AUC of 0.7381, sensitivity of 0.6905, specificity of 0.6675, and accuracy of 0.6782 [49]. Another developed predictive models for periodontal disease in a Korean population using LR, support vector machines (SVM), RF, XGBoost, and neural networks (NN). The optimal model (XGBoost) demonstrated a test set AUC value of 0.823 [50]. Yet another employed mixed-effects logistic regression (MELR), mixed-effects decision tree (MEDT), recurrent neural network (RNN), and mixed-effects support vector machine (MESVM) models to identify high-risk individuals with severe periodontitis among Thai adults [51]. The best-performing model, MELR, achieved an AUC of 0.983, with a 95% CI of 0.977 to 0.989. Compared with these studies (the details are shown in Table 3), this work has the following differences: (1) We utilized LASSO regression to screen variables, improving model parsimony and mitigating overfitting risks. (2) SHAP analysis enabled model interpretability through global feature evaluation and pediatric gingivitis risk factors. (3) Unlike previous studies focusing on adults or adolescents, our model specifically targets children aged 6 to 12, a pivotal period for early intervention before pubertal hormonal shifts amplify disease susceptibility.

**Table 3:** Comparison of this study with previously reported studies using ML techniques for periodontal disease

Aspect	Study population	Disease focus	Feature extraction	Data analysis method	Model interpretation	Performance metrics
Chen et al. [49] (2020)	12-year-old children in Sichuan Province, China	Gingival bleeding	Univariate analysis (Pearson’s Chi-square test)	LR	LR analysis	AUC: 0.7381
Woosun Beak et al. [50] (2024)	Korean adults	Periodontal disease	Select potential risk factors associated with periodontitis, followed by Pearson correlation analysis.	LR, SVM, RF, XGBoost, and NN	The feature importance	AUC: 0.823 (XGBoost)
Htun Teza MSc et al. [51] (2023)	Thai adults	Severe periodontitis	Univariate analysis followed by stepwise forward multivariate analysis	MELR, RNN, MESVM, and MEDT	Not reported	AUC: 0.983 (MELR)
This study	Schoolchildren aged 6– 12 years in Lanzhou, China	Gingivitis	LASSO regression	LR, DT, RF, LightGBM, KNN, and XGBoost	SHAP-based global feature importance and individualized risk visualization	AUC: 0.909 (RF)

The observed AUC slightly declines during external validation may be partly explained by seasonal influences. Specifically, the cohort was validated in March—a peak season for pediatric respiratory infections in Lanzhou [52–54]. Such seasonal outbreaks have been shown to alter healthcare utilization patterns, often diverting attention from non-urgent preventive services [55, 56]. This shift could attenuate the predictive power of the “regular dental checkups” variable in our model. Heterogeneity in team composition also cannot be ruled out as a contributing factor. Notably, the model maintained strong diagnostic utility (AUC >0.800), supporting its robustness amid real-world variability.

However, several limitations should be acknowledged. First, our cross-sectional design inherently limits causal inference and longitudinal risk prediction. The model identifies children with current high-risk profiles rather than predicting future gingivitis onset. For instance, we cannot distinguish whether infrequent brushing causes gingivitis or if pre-existing gingivitis and associated discomfort lead to reduced brushing. While this aligns with the goal of community-level screening for immediate intervention, future longitudinal cohort studies that track incident gingivitis cases are necessary to validate predictors of disease progression and refine risk trajectories. Second, the model’s reliance on self-reported proxies, while crucial for its scalability and feasibility in a public health context, means it does not capture the underlying biological state of the periodontium. It cannot assess the specific composition of the plaque microbiome or the presence of particular periodontal pathobions, which are critical in disease pathogenesis [57–59]. Future studies that integrate such biological data with self-reported predictors could powerfully enhance model precision and biological plausibility. Third, the temporal validation cohort—though independent—was sourced from the same city, potentially overfitting to local socioeconomic patterns. This restricts extrapolation to populations with divergent cultural practices (e.g., dietary habits, oral hygiene norms). Furthermore, while the class distribution in our dataset was nearly balanced, this might not be the case in other populations. The model’s performance, particularly the clinical interpretation of a positive or negative prediction, is inherently dependent on disease prevalence and could vary in settings with substantially higher or lower gingivitis prevalence. Future multi-center validations should specifically test the model in socio-culturally diverse and imbalanced populations to assess robustness across different epidemiological contexts. Additionally, questionnaire data are subject to social desirability bias (e.g., overreporting of brushing frequency) and recall bias, particularly relevant for younger children relying on parental proxies. Although the instrument demonstrated strong content validity and feasibility, it was not assessed for test–retest reliability. A future reliability study administering the questionnaire to a sub-sample of participants after a 2-week interval would help quantify and mitigate measurement error.

## Conclusion

This study validates an interpretable RF model for community-based pediatric gingivitis screening. Demonstrating strong discriminatory power (training AUC: 0.991; testing AUC: 0.909) and generalizability (external validation AUC: 0.824), the tool relies solely on self-reported questionnaires, reducing the need for universal clinical exams through selective referral. Key predictors—brushing frequency, age, regular dental checkups, brushing time, gingival bleeding during brushing, and annual income—identified via SHAP analysis enable targeted interventions. This model shows promise as a potential primary screening tool in resource-constrained settings, allowing for the early detection of individuals predisposed to gingivitis and enabling targeted preventive interventions in school-based programs. Future research should focus on validating this model in multi-center, diverse populations and integrating it into school-based oral health programs to assess its real-world efficacy and practical impact.

## Supplementary Information

Supplementary Material 1: Self-administered questionnaire for assessing gingivitis risk factors in schoolchildren aged 6–12 years

Supplementary Material 2: Supplementary Table 1. Final optimal hyperparameters for the machine learning algorithms used to predict gingivitis in children aged 6–12

Supplementary Material 3: Supplementary Table 2. Coefficients of variables retained in LASSO regression

Supplementary Material 4: Supplementary Table 3. Baseline characteristics of the validation cohort

## Data Availability

Data are available from the corresponding author upon reasonable request. The data are not publicly available due to privacy or ethical restrictions.

## Abbreviations

SHAP: SHapley Additive exPlanations
LightGBM: Light gradient boosting machine
RF: Random forest
LR: Logistic regression
XGBoost: eXtreme gradient boosting
DT: Decision tree
KNN: K-nearest neighbors
LASSO: Least Absolute Shrinkage and Selection Operator
ROC: Receiver operating characteristic
AUC: Area under the curve
ML: Machine learning
MEDT: Multi-enhanced decision tree
MELR: Multi-enhanced logistic regression
MESVM: Multi-enhanced support vector machine
NN: Neural network
RNN: Recurrent neural network
