# Supplementary material for: Predicting plaque-gingivitis risk in schoolchildren using an interpretable machine learning model: a cross-sectional study
Source: BMC Oral Health. 2025 Dec 15;25:1910. doi: 10.1186/s12903-025-07245-y (PMC12706944; doi:10.1186/s12903-025-07245-y)
Supplement: Supplementary file 1 — Supplementary Material 1: Self-administered questionnaire for assessing gingivitis risk factors in schoolchildren aged 6–12 years [file 12903_2025_7245_MOESM1_ESM.docx]

**Self-administered questionnaire for assessing gingivitis risk factors in schoolchildren aged 6**–**12 years.**

**Instructions:**Please complete this questionnaire about your child’s oral health.**Sections A, B, and D are to be completed by a parent or guardian. Section C is designed to be answered by the child themselves, with assistance from the interviewer if needed.**

- Name: ______
- Date of Birth: _____
- Sex: ______
- Height (cm): ______
- Weight (kg): ______
- Fill in the Date: ______

***Section A: Demographic and Socioeconomic Background***

**A1: Family Residence Area:**

- Urban
- Rural

**A2: Is your child the only child in the family?**

- Yes
- No

**A3: Father’s Highest Educational Level:**

- ≤ 9 years (Junior high school or below)
- 10–12 years (Senior high school)
- ≥ 13 years (College or university degree and above)

**A4: Mother’s Highest Educational Level:**

- ≤ 9 years (Junior high school or below)
- 10–12 years (Senior high school)
- ≥ 13 years (College or university degree and above)

**A5: Annual Family Income (CNY):**

- ≤ 50,000
- 50,000–100,000
- 100,000–200,000
- 200,000–400,000
- ≥ 400,000

***Section B: Oral Hygiene Behaviors***

**B1. How often does your child brush their teeth?**

- Twice a day or more
- Once a day
- Not every day

**B2. How long does your child usually brush their teeth for each time?**

- ≥ 3 minutes
- 1–3 minutes
- ≤ 1 minute

**B3. Does your child use dental floss?**

- Yes
- No

**B4. Does your child use fluoride toothpaste?**

- Yes
- No

**B5. Does your child rinse their mouth with water after meals?**

- Yes
- No

**B6. How often is your child’s toothbrush replaced?**

- ≤ Every 3 months
- > Every 3 months

**B7. Does your child’s gum bleed during tooth brushing?**

- Never
- Sometimes
- Frequently

**B8. Do you, as a parent, supervise your child’s tooth brushing every day?**

- Yes
- No

**B9. Does your child have regular annual dental check-ups?**

- Yes
- No

**B10. How frequently does your child consume sugary foods or drinks (e.g., candy, cake, soda)?**

- Once a day or more
- Two to six times a week
- Once a week or less

***Section C: Oral Health Knowledge (To be answered by the CHILD)***

**C1. Do you think it is normal if your gums bleed when you brush your teeth?**

- Yes
- No
- Unclear

**C2. Do you think brushing your teeth properly can help prevent your gums from bleeding?**

- Yes
- No
- Unclear

***Section D: Undesirable Oral Habits***

**D1. Does your child have a habit of unilateral chewing (chewing on only one side of the mouth)?**

- Yes
- No

**D2. Does your child have a habit of mouth breathing (breathing through the mouth during the day or at night)?**

- Yes
- No

**Thank you for your participation and valuable time!**
