# Supplementary material for: Predicting plaque-gingivitis risk in schoolchildren using an interpretable machine learning model: a cross-sectional study
Source: BMC Oral Health. 2025 Dec 15;25:1910. doi: 10.1186/s12903-025-07245-y (PMC12706944; doi:10.1186/s12903-025-07245-y)
Supplement: Supplementary file 2 — Supplementary Material 2: Supplementary Table 1. Final optimal hyperparameters for the machine learning algorithms used to predict gingivitis in children aged 6–12 [file 12903_2025_7245_MOESM2_ESM.docx]

**Supplementary Table 1.** Final optimal hyperparameters for the machine learning algorithms used to predict gingivitis in children aged 6–12.

| Model *(R packages)* | Hyperparameter | Final value |
| --- | --- | --- |
| *XGBoost (xgboost)* | objective | "binary:logistic" |
|  | eta | 0.10 |
|  | nrounds | 500 |
|  | max_depth | 2 |
|  | min_child_weight | 1 |
|  | gamma | 0.10 |
|  | subsample | 0.80 |
|  | colsample_bytree | 0.80 |
|  | lambda | 1 |
| LightGBM *(lightgbm)* | lambda_l1 | 1 |
|  | lambda_l2 | 1 |
|  | learning_rate | 0.10 |
|  | num_iterations | 500 |
|  | max_depth | 3 |
|  | num_leaves | 8 |
| RF *(randomForest)* | ntree | 500 |
|  | mtry | 2 |
|  | nodesize | 5 |
| KNN *(kknn)* | k | 9 |
|  | distance | 2 |
|  | kernel | "optimal" |
| DT *(rpart)* | cp | 0.00410509 |
|  | maxdepth | 10 |
|  | minsplit | 3 |

The final LR classifier was refitted with *glm* (binomial) on the selected predictors.
