# Supplementary material for: Predicting plaque-gingivitis risk in schoolchildren using an interpretable machine learning model: a cross-sectional study
Source: BMC Oral Health. 2025 Dec 15;25:1910. doi: 10.1186/s12903-025-07245-y (PMC12706944; doi:10.1186/s12903-025-07245-y)
Supplement: Supplementary file 3 — Supplementary Material 3: Supplementary Table 2. Coefficients of variables retained in LASSO regression [file 12903_2025_7245_MOESM3_ESM.docx]

**Supplementary Table 2.** Coefficients of variables retained in LASSO regression.

| Importance of variates (ordered) | Estimate | Lambda |
| --- | --- | --- |
| Brushing frequency | 1.0299699 |  |
| Regular dental checkups | 0.9811771 |  |
| Brushing time | 0.7762892 |  |
| Floss daily | 0.4277576 |  |
| Bleeding from brushing | 0.3907766 |  |
| Age | 0.1363992 |  |
| Fluoride toothpaste | 0.1099413 |  |
| Mouth breathing | -0.1702517 |  |
| Father's education level | -0.280915 |  |
| Annual income | -0.3786905 |  |
| Mother's education level | -0.3861112 |  |
|  |  | 0.02261307 |
