# Supplementary material for: Predicting plaque-gingivitis risk in schoolchildren using an interpretable machine learning model: a cross-sectional study
Source: BMC Oral Health. 2025 Dec 15;25:1910. doi: 10.1186/s12903-025-07245-y (PMC12706944; doi:10.1186/s12903-025-07245-y)
Supplement: Supplementary file 4 — Supplementary Material 4: Supplementary Table 3. Baseline characteristics of the validation cohort [file 12903_2025_7245_MOESM4_ESM.docx]

**Supplementary Table 3.** Baseline characteristics of the validation cohort.

| Variable | N (120) | Normal (n = 61) n (%) | Gingivitis (n = 59) n (%) | *p - value* |
| --- | --- | --- | --- | --- |
| ***Socio-demographic characteristics*** |  |  |  |  |
| Age (year) |  |  |  | < 0.001 |
| 6 | 10 (8.3%) | 9 (14.8%) | 1 (1.7%) |  |
| 7 | 19 (15.8%) | 16 (26.2%) | 3 (5.1%) |  |
| 8 | 12 (10.0%) | 11 (18.0%) | 1 (1.7%) |  |
| 9 | 19 (15.8%) | 9 (14.8%) | 10 (16.9%) |  |
| 10 | 21 (17.5%) | 8 (13.1%) | 13 (22.0%) |  |
| 11 | 18 (15.0%) | 4 (6.6%) | 14 (23.7%) |  |
| 12 | 21 (17.5%) | 4 (6.6%) | 17 (28.8%) |  |
| Sex |  |  |  | 0.869 |
| Male | 54 (45.0%) | 27 (44.3%) | 27 (45.8%) |  |
| Female | 66 (55.0%) | 34 (55.8%) | 32 (54.2%) |  |
| Area |  |  |  | 0.475 |
| Urban | 67 (55.8%) | 36 (59.0%) | 31 (52.5%) |  |
| Rural | 53 (44.2%) | 25 (41.0%) | 28 (47.5%) |  |
| BMI |  |  |  | 0.539 |
| Underweight | 16 (13.3%) | 7 (11.5%) | 9 (15.3%) |  |
| Normal | 73 (60.8%) | 35 (57.4%) | 38 (64.4%) |  |
| Obesity | 27 (22.5%) | 16 (26.2%) | 11 (18.6%) |  |
| Overweight | 4 (3.3%) | 3 (4.9%) | 1 (1.7%) |  |
| If only child |  |  |  | 0.897 |
| Yes | 40 (33.3%) | 20 (32.8%) | 20 (33.9%) |  |
| No | 80 (66.7%) | 41 (67.2%) | 39 (66.1%) |  |
| Father’s education level |  |  |  | < 0.001 |
| ≤ 9 years | 26 (21.7%) | 6 (9.8%) | 20 (33.9%) |  |
| 10–12 years | 14 (11.7%) | 5 (8.2%) | 9 (15.3%) |  |
| ≥ 13 years | 80 (66.7%) | 50 (82.0%) | 30 (50.8%) |  |
| Mother’s education level |  |  |  | < 0.001 |
| ≤ 9 years | 28 (21.9%) | 7 (11.5%) | 21 (35.6%) |  |
| 10–12 years | 14 (18.8%) | 3 (4.9%) | 11 (18.6%) |  |
| ≥ 13 years | 78 (65.0%) | 51 (83.6%) | 401 (45.8%) |  |
| Annual family income (yuan) |  |  |  | 0.153 |
| ≤ 50000 | 9 (7.5%) | 6 (9.8%) | 3 (5.1%) |  |
| 50000–100000 | 64 (53.3%) | 26 (42.6%) | 38 (64.4%) |  |
| 100000–200000 | 22 (18.3%) | 12 (19.7%) | 10 (16.9%) |  |
| 200000–400000 | 21 (17.5%) | 14 (23.0%) | 7 (11.9%) |  |
| ≥ 400000 | 4 (3.3%) | 3 (4.9%) | 1 (1.7%) |  |
| ***Oral health behaviors*** |  |  |  |  |
| Brushing frequency |  |  |  | < 0.001 |
| Twice a day or more | 56 (46.7%) | 40 (65.6%) | 16 (27.1%) |  |
| Once a day | 54 (45.0%) | 20 (32.8%) | 34 (57.6%) |  |
| Not everyday | 10 (8.3%) | 1 (1.6%) | 9 (15.3%) |  |
| Brushing time |  |  |  | 0.076 |
| ≥ 3 min | 31 (25.8%) | 21 (34.4%) | 10 (16.9%) |  |
| 1–3 min | 80 (66.7%) | 37 (60.7%) | 43 (72.9%) |  |
| ≤ 1 min | 9 (7.5%) | 3 (4.9%) | 6 (10.2%) |  |
| Floss daily |  |  |  | 0.062 |
| Yes | 15 (12.5%) | 11 (18.0%) | 4 (6.8%) |  |
| No | 105 (87.5%) | 50 (82.0%) | 55 (93.2%) |  |
| Fluoride toothpaste use |  |  |  | 0.074 |
| Yes | 5 (4.2%) | 5 (8.2%) | 0 (0.0%) |  |
| No | 115 (95.8%) | 56 (91.8%) | 59 (100.0%) |  |
| Rinsing after meals |  |  |  | 0.752 |
| Yes | 29 (24.2%) | 14 (23.0%) | 15 (25.4%) |  |
| No | 91 (75.8%) | 47 (77.0%) | 44 (74.6%) |  |
| Toothbrush change frequency |  |  |  | < 0.001 |
| ≤ 3 months | 68 (56.7%) | 25 (41.0%) | 43 (72.9%) |  |
| > 3 months | 52 (43.3%) | 36 (59.0%) | 16 (27.1%) |  |
| Bleeding from brushing |  |  |  | 0.396 |
| Never | 62 (51.7%) | 35 (57.4%) | 27 (45.8%) |  |
| Sometimes | 54 (45.0%) | 24 (39.3%) | 30 (50.8%) |  |
| Frequently | 4 (3.3%) | 2 (3.3%) | 2 (3.4%) |  |
| Your parents will supervise your tooth brushing every day |  |  |  | 0.302 |
| Yes | 40 (33.3%) | 23 (37.7%) | 17 (28.8%) |  |
| No | 80 (66.7%) | 38 (62.3%) | 42 (71.2%) |  |
| Regular annual dental checkups |  |  |  | 0.002 |
| Yes | 45 (37.5%) | 31 (50.8%) | 14 (23.7%) |  |
| No | 75 (62.5%) | 30 (49.2%) | 45 (76.3%) |  |
| Consumption of sugars and sweet foods |  |  |  | 0.007 |
| Once a day or more | 17 (14.2%) | 14 (23.0%) | 3 (5.1%) |  |
| Two to six times a week | 73 (60.8%) | 30 (49.2%) | 43 (72.9%) |  |
| Once a week or less | 30 (25.0%) | 17 (27.9%) | 13 (22.0%) |  |
| ***Oral health knowledge*** |  |  |  |  |
| Bleeding from brushing is normal |  |  |  | 0.001 |
| Yes | 31 (25.8%) | 11 (18.0%) | 20 (33.9%) |  |
| No | 65 (54.2%) | 43 (70.5%) | 22 (37.3%) |  |
| Unclear | 24 (20.0%) | 7 (11.5%) | 17 (28.8%) |  |
| Brushing prevents bleeding gums |  |  |  | 0.434 |
| Yes | 17 (14.2%) | 9 (14.8%) | 8 (13.6%) |  |
| No | 46 (38.3%) | 20 (32.8%) | 26 (44.1%) |  |
| Unclear | 57 (47.5%) | 32 (52.5%) | 25 (42.4%) |  |
| *Oral bad habits* |  |  |  |  |
| Unilateral chewing |  |  |  | > 0.99 |
| Yes | 14 (11.7%) | 7 (11.5%) | 7 (11.9%) |  |
| No | 106 (83.3%) | 54 (88.5%) | 52 (88.1%) |  |
| Mouth breathing |  |  |  | 0.947 |
| Yes | 13 (10.8%) | 6 (9.8%) | 7 (11.9%) |  |
| No | 107 (89.2%) | 55 (90.2%) | 52 (88.1%) |  |
